# Protothecosis in the mucosa of the pharynx mimicking pharyngeal cancer in an immunocompetent individual: a case report

**DOI:** 10.1186/s12941-022-00495-6

**Published:** 2022-02-14

**Authors:** Marie Yamashita, Mahoko Ikeda, Ito Kato, Yuki Ohama, Mizuo Ando, Masako Ikemura, Daisuke Jubishi, Yoshiaki Kanno, Koh Okamoto, Takashi Umeyama, Shigeki Nakamura, Yoshitsugu Miyazaki, Shu Okugawa, Kyoji Moriya

**Affiliations:** 1 Department of Infectious Diseases, The University of Tokyo Hospital, 7-3-1, Hongo, Bunkyo-ku, Tokyo, 113-8655 Japan; 2 Department of Infection Control and Prevention, The University of Tokyo Hospital, 7-3-1, Hongo, Bunkyo-ku, Tokyo, 113-8655 Japan; 3 Department of Clinical Laboratory, National Hospital Organization Tokyo Medical Center, 2-5-1, Higashigaoka, Meguro-ku, Tokyo, 152-8902 Japan; 4 Department of Otorhinolaryngology and Head and Neck Surgery, The University of Tokyo Hospital, 7-3-1, Hongo, Bunkyo-ku, Tokyo, 113-8655 Japan; 5 Department of Pathology, The University of Tokyo Hospital, 7-3-1, Hongo, Bunkyo-ku, Tokyo, 113-8655 Japan; 6 Department of Chemotherapy and Mycoses, National Institute of Infectious Diseases, 1-23-1, Toyama, Shinjuku-ku, Tokyo, 162-8640 Japan; 7 Department of Microbiology, Tokyo Medical University, 6-1-1, Shinjuku, Shinjuku-ku, Tokyo, 160-8402 Japan

**Keywords:** *Prototheca wickerhamii*, Protothecosis, Larynx, *Cryptococcus*, Matrix-assisted laser desorption/ionization time-of-flight mass spectrometry

## Abstract

**Background:**

Protothecosis is a rare infection in humans and animals caused by the achlorophyllic algae *Prototheca* species. More than half of the protothecosis cases are cutaneous infections, and most cases are observed in immunocompromised individuals.

**Case presentation:**

We report a case of *Prototheca wickerhamii* infection in the mucosa of the pharynx in a 53-year-old immunocompetent woman with an incidentally found mass lesion at the left tongue base. Histopathological findings of the mass lesion suggested cryptococcosis, but *P. wickerhamii* was identified from the oropharynx scrape culture based on DNA sequencing. After surgical resection, fosfluconazole treatment was initiated, and subsequently, treatment was switched to topical amphotericin B. The residual mass lesion did not deteriorate during the 4-month antifungal treatment and 1-year observational period.

**Conclusions:**

*Prototheca* species can be easily misdiagnosed as yeasts because of their morphological and pathological similarities. *Prototheca*, in addition to *Cryptococcus* should be considered if slow-growing, large Gram-positive organisms are encountered. Lactophenol cotton blue staining of the colony helps distinguish these organisms. Further study is needed to determine the appropriate treatment according to the infection focus.

## Background

Protothecosis is a rare infection caused by *Prototheca* species in both humans and animals. These organisms are achlorophyllic algae that are ubiquitous in the environment and animal intestinal flora. The genus *Prototheca* is divided into eight species, and the most common causative species of human infection are *Prototheca wickerhamii* and *Prototheca zopfi* [1]. Since the first case of human protothecosis in 1964, approximately 200 cases have been reported [2–4]. The clinical forms of protothecosis are classified into three types: cutaneous disease, olecranon bursitis, and disseminated disease [3, 5, 6]. More than half of the cases are cutaneous infections, and immunosuppression is a predisposing factor for human protothecosis [2–4].

We report a case of *P. wickerhamii* infection in the pharynx of an immunocompetent patient, successfully treated with a combination of surgical resection and medical therapy.

## Case presentation

A 53-year-old Japanese woman with no significant medical history other than chronic gastritis, diagnosed by upper gastrointestinal endoscopy 6 years previously, presented to our hospital with a mass in the larynx that appeared to be malignant. She had a 1-year history of a dull feeling in her throat and cough. Three months earlier, she had been diagnosed with anisakiasis at a local clinic and had been incidentally found to have mass lesion of approximately 7-mm in diameter at the left tongue base, by upper gastrointestinal endoscopy. One month earlier, follow-up nasopharyngoscopy had revealed no changes in the mass lesion, and an endoscopic biopsy had been performed. Squamous cell carcinoma was suspected pathologically, and the patient was referred to the department of otorhinolaryngology at our hospital for further evaluation.

**Fig. 1:**
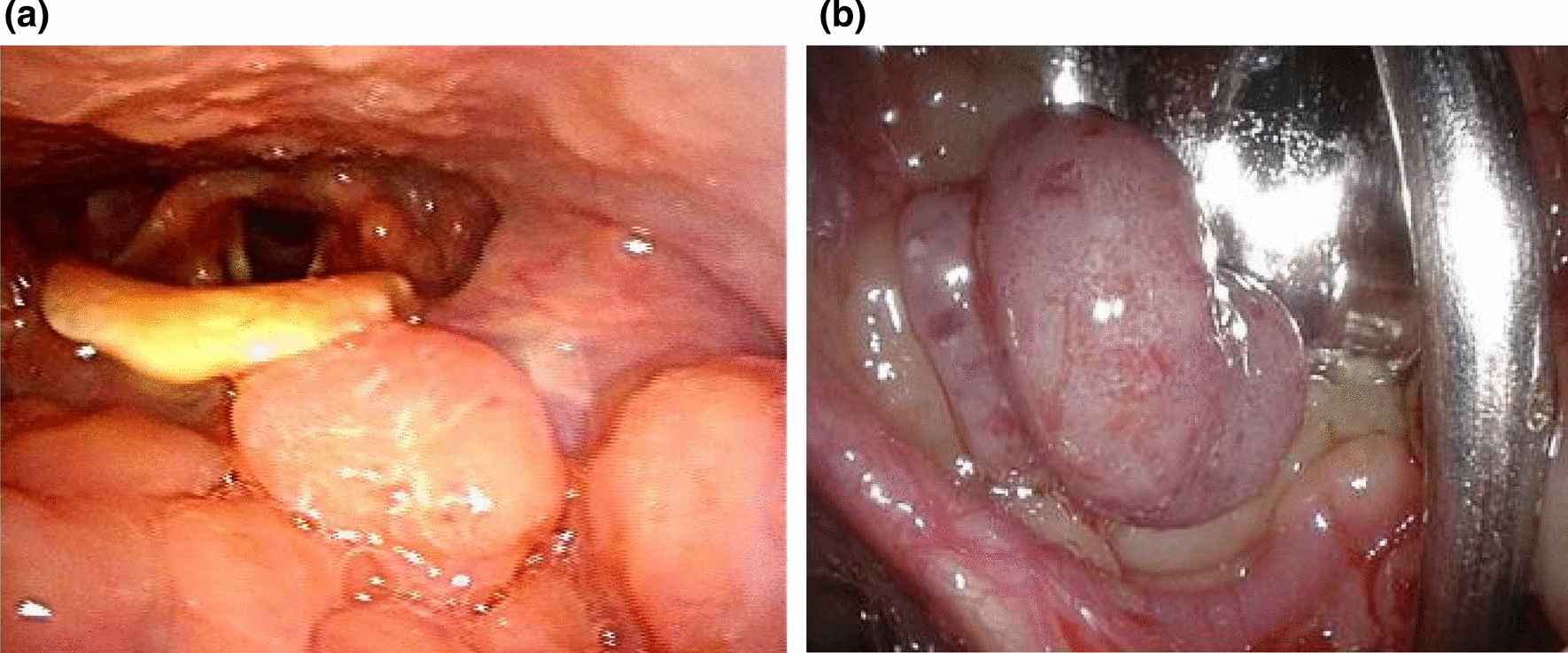
Macroscopic appearance of protothecosis in the pharyngeal mucosa. ** a** Endoscopic image taken at our outpatient clinic showing a polypoid mass at the left tongue base. ** b** Intraoperative close-up image showing a reddish, smooth, and pedunculated mass without adjacent dysplastic mucosa

An endoscopic biopsy was also performed in our outpatient clinic (Fig. 1a), but the biopsy specimens only showed atypical epithelium, and the scrape culture was negative. Intravenous contrast-enhanced computed tomography (CT) of the neck and thorax was unremarkable, except for bilateral cervical lymphadenopathy.

**Fig. 2:**
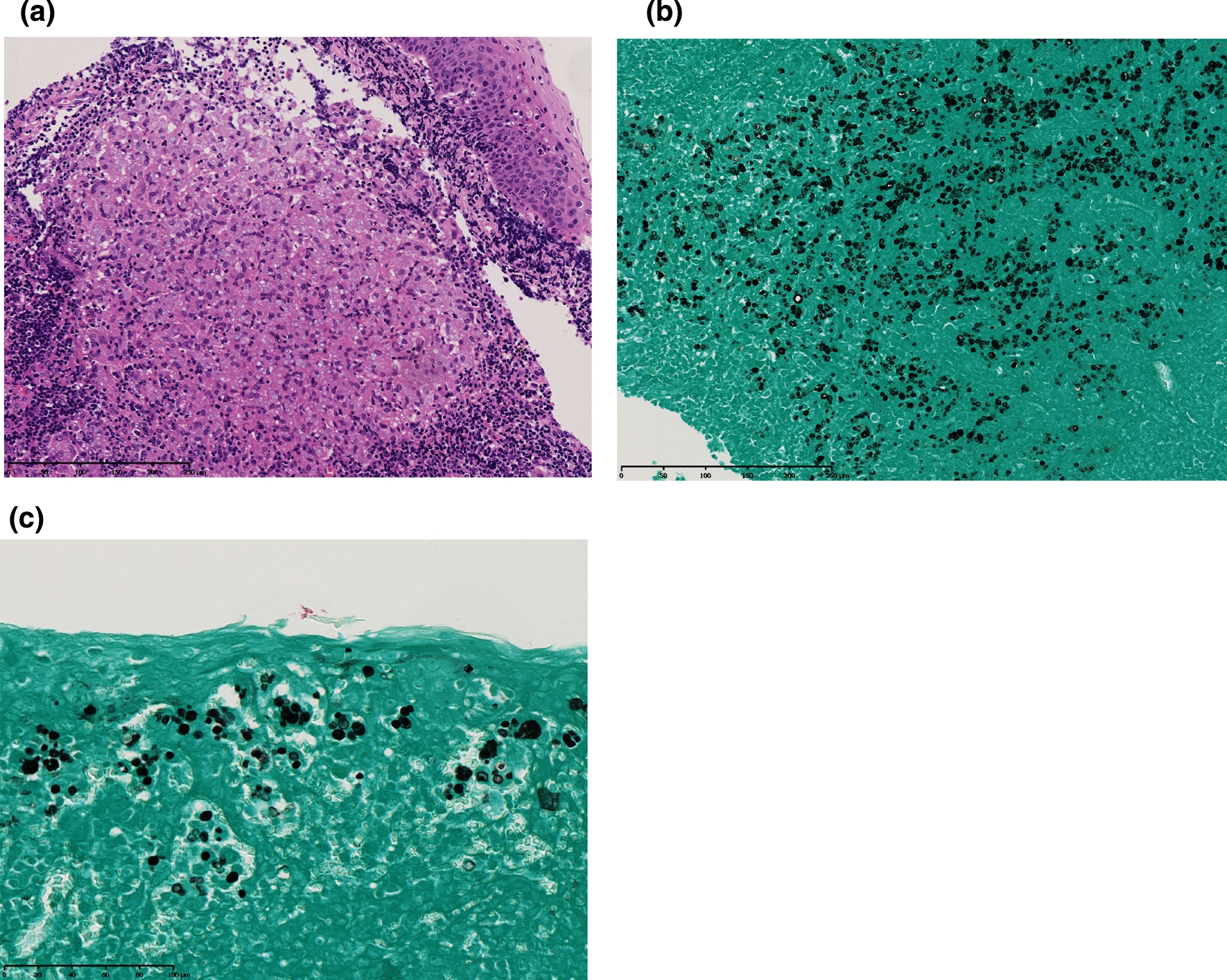
Histopathological findings of protothecosis in the excision biopsy specimen. ** a** Hematoxylin and Eosin staining of the lesion showing granulomatous inflammation. ** b**, **c** Grocott–Gomori methenamine silver staining of mass lesion biopsy specimen showing encapsulated yeast-like organisms

For further evaluation, an excision biopsy under general anesthesia was performed (Fig. 1b). Histopathological examination of a hematoxylin and eosin-stained biopsy specimen showed granulomatous tissue consisting mainly of histiocytes and multinucleated giant cells (Fig. 2a). Some histiocytes had phagocytized the encapsulated yeast-like organisms that were invading the epithelium. There were also scant neutrophils, but no micro-abscesses were found. The walls of the mass were positive on staining with Grocott’s methenamine silver (Fig. 2b and c). These findings suggested cryptococcosis; therefore, she was referred to the Department of Infectious Disease for the treatment of the residual mass lesion.

On physical examination, the patient was afebrile, and her vital signs were normal. Head and neck examination revealed no enlarged lymph nodes, and no meningeal signs. Examination of the pharynx revealed no pharyngeal edema or exudate. She had no skin lesions. The blood test results were unremarkable. An HIV antibody/antigen combination test result was negative. A neutrophil function test was not performed because she did not have a history of recurrent or severe bacterial infection, suggesting that her neutrophil function was normal. Chest CT revealed no pulmonary findings of note. A serum *Cryptococcus* antigen test (Bio-Medical Laboratories, Inc.), using a latex agglutination method was negative. Serum beta D glucan was not measured. Based on these findings and the histopathology, she was provisionally diagnosed with possible non-meningeal, non-pulmonary cryptococcosis. The scrape culture of the residual lesion at the base of the tongue was repeated, and then fosfluconazole treatment (6 mg/kg bodyweight/day) was initiated as treatment for localized cryptococcosis.

**Fig. 3:**
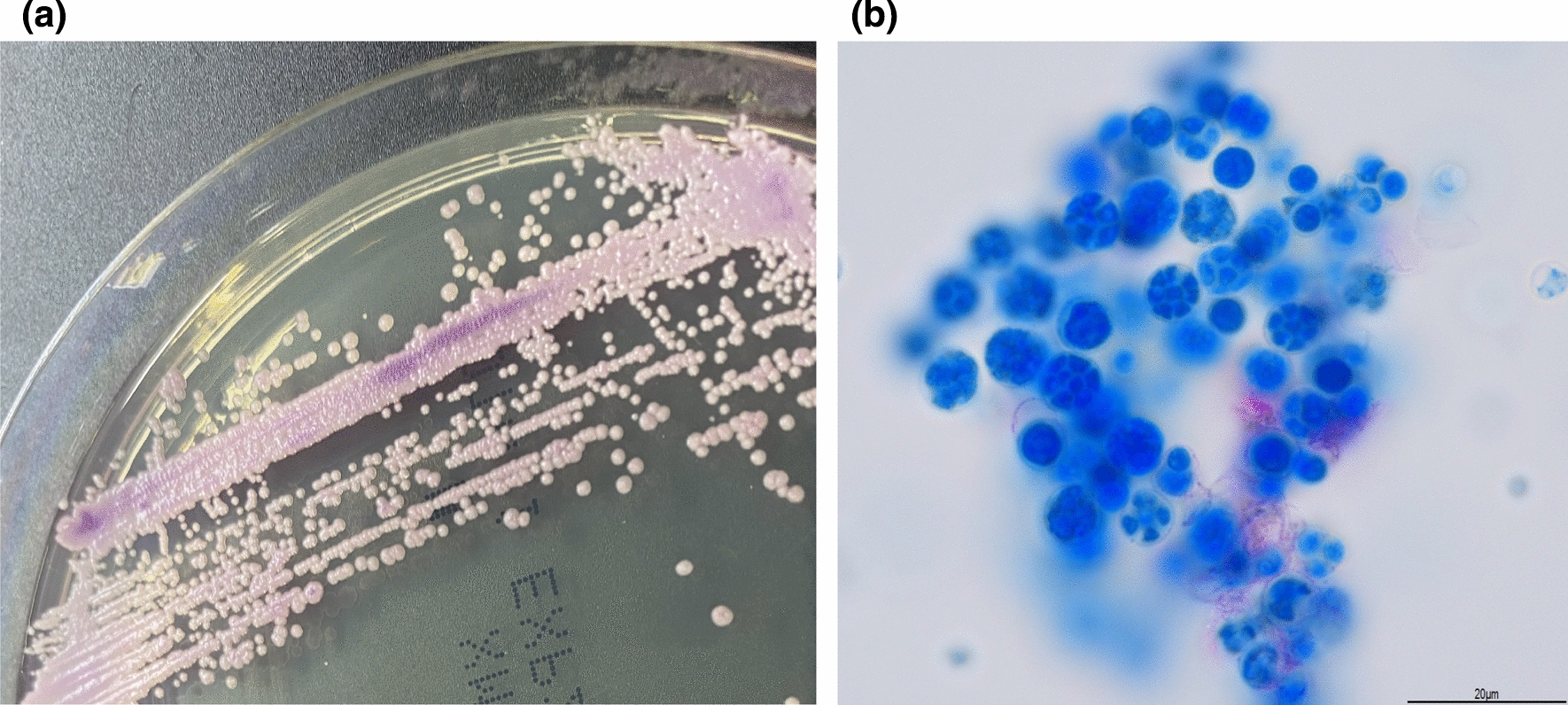
Microbiological findings of the *Prototheca* isolate. ** a** Colony appearance of the *Prototheca* isolate grew on XM-Candida agar plate (Nissui Pharmaceutical Co., Ltd., Tokyo, Japan) at 35 °C in aerobic conditions for 3 days. ** b** Lactophenol cotton blue staining of the *Prototheca* colony showing tightly packed endospores within a sporangium. (Pictured over cover glass at 1000× magnification using an oil immersion lens)

After 3 days of incubation of the separation culture that targeted *Cryptococcus* from the scrape specimen, white to pale purple-colored small colonies grew on the XM-Candida agar plate (Nissui Pharmaceutical Co., Ltd., Tokyo, Japan) cultured at 35 °C in aerobic conditions (Fig. 3a). The VITEK® 2 COMPACT Microbial Detection System (version 8.01 database: SYSMEX bioMérieux Co., Ltd., Tokyo, Japan) based on the biochemical reaction method with yeast identification card identified the colonies as *P. wickerhamii.* Matrix-assisted laser desorption/ionization time-of-flight mass spectrometry (MALDI-TOF MS, using the MALDI Biotyper version 4.0.0.1 database; Bruker Daltonik, Germany) did not identify the colonies initially, but in the re-examination, it identified the colonies as *P. wickerhamii* with low probability (score 1.451). Lactophenol cotton blue staining of the colony revealed tightly packed endospores within the sporangia distinctive of *P. wickerhamii* [7] (Fig. 3b).

DNA was extracted and polymerase chain reaction (PCR) method was performed using primers to amplify the internal transcribed spacer region and the D1/D2 domain of the large subunit ribosomal DNA gene. Sequence analysis of the amplicons showed no significant results, suggesting genetic polymorphism. Cloning was performed, and base sequences showing high homology with *P. wickerhamii* genes were detected.

Therefore, a diagnosis of laryngeal protothecosis was established. The minimum inhibitory concentration (MIC) results using the Frozen Plate for Antifungal Susceptibility Testing of Yeasts, Eiken (Eiken Chemical Co., Ltd., Tokyo) were as follows: amphotericin B, 1 µg/mL; fluconazole, > 64 µg/mL; itraconazole, 4 µg/mL; voriconazole, 1 µg/mL; miconazole, > 16 µg/mL; flucytosine, > 64 µg/mL; and micafungin, > 16 µg/mL. Empiric fosfluconazole treatment was discontinued after 10 weeks because the size of the residual mass lesion did not change. Amphotericin B syrup (1 mL, 4 times a day) was initiated and continued for 6 weeks instead of intravenous amphotericin B treatment because the patient was asymptomatic and could not take time off from work to be admitted to hospital for intravenous amphotericin B treatment. Although we considered additional and definitive resection after the patient was diagnosed with *Prototheca* infection, we decided against it because we anticipated that it would be difficult to remove the lesion with safety margins because the vertical margin was not clearly determined on macroscopic examination, and resection carried a risk of causing difficulties with speech and swallowing. The residual mass lesion did not deteriorate during the antifungal treatment or the post-treatment one-year follow-up period.

## Discussion and conclusions

Protothecosis has been classified into three types of clinical forms: cutaneous infections, olecranon bursitis, and disseminated infections. More than half of protothecosis cases are cutaneous infections [3, 5, 6]. The majority of cases occur in immunosuppressed individuals [3, 4], while olecranon bursitis can occur in immunocompetent individuals after some types of penetrating trauma to the elbow [6]. The main underlying conditions of protothecosis are local or systemic steroid use, hematologic malignancy or cancer, diabetes mellitus, acquired immunodeficiency syndrome, solid organ transplantation, alcoholism, and peritoneal dialysis [3]. We describe a rare case of protothecosis in the pharyngeal mucosa of an immunocompetent patient.

Although there have been approximately 200 reports of protothecosis [2], to our knowledge, there have been only two previous reports of protothecosis in the field of otorhinolaryngology: a nasopharyngeal ulceration complicating prolonged endotracheal intubation in 1992 [8] and protothecosis of the larynx [9]. The laryngeal infection occurred in an immunocompetent individual, but a branchiogenic cyst was observed close to the site of the infection, and it was suspected that the inflamed cyst might have provided a portal of entry for *Prototheca* species. Unlike the two previous cases, our patient did not have any pre-existing mucosal defects or anatomical abnormalities. She had undergone upper gastrointestinal endoscopy 6 years before the diagnosis but there have been no reports of *Prototheca* algae being introduced to the pharynx by endoscopy; thus, the source of the infection is unclear. The patient’s blood test results and medical history did not suggest an immune-compromised state; however, it has been reported that qualitative factors may play a greater role than quantitative factors in neutrophil defense against *P. wickerhamii* [10]. Thus, it is difficult to evaluate the actual level of immunity against *Prototheca* species.

The diagnosis of protothecosis is conventionally based on morphological and biochemical tests of the isolated organism and histopathological tests of the affected tissues [1, 3]. The colony characteristics of *Prototheca* species are similar to those of yeasts such as *Candida* species or *Cryptococcus* species [5, 11] and there have been case reports that mistakenly identified *Prototheca* species as yeasts [12–14]. The morphological characteristics of *Prototheca* species are due to their life cycle, where they reproduce asexually by releasing numerous sporangiospores [5, 11]. They can be distinguished from yeasts if typical morula forms containing sporangiospores are observed with lactophenol cotton blue staining [12]. However, we were unable to isolate *P. wickerhamii* from the oropharynx scrape culture during the initial attempt. There are two possible reasons for our failure to isolate *Prototheca* species: First, they are easily overgrown by bacteria when the culture is taken from contaminated sites, such as the pharynx. Second, most *Prototheca* species require incubation at 30 °C for 72 h, whereas some slow-growing strains require incubation at 25 °C for up to 8 days; thus, they can be missed using standard culture methods [3, 11].

In the histopathological examination, characteristic morula forms are helpful for diagnosing protothecosis, but there can be a lack of such findings due to the period of their life cycle. The external capsule of *Prototheca* species and the wall of yeasts stain positive with Grocott’s methenamine silver and periodic acid-Schiff stains [15]. Thus, they may be mistaken for *Candida* or *Cryptococcus* species, as we experienced.

As discussed above, *Prototheca* species can be easily misdiagnosed as yeasts because of their morphological and pathologic similarities. In our case, VITEK2 and MALDI-TOF MS testing were useful for making the diagnosis. Although the results are not reproducible, we established a differential diagnosis of protothecosis. Recently, molecular characterization of ribosomal DNA has been exploited for intraspecies identification of *Prototheca* species. Previous studies have shown that comprehensive analysis by PCR of the internal transcribed spacer region and the large subunit D1/D2 domain is useful for species identification [16, 17]. In this case, we confirmed the diagnosis of protothecosis based on DNA sequencing, combined with morphological, histopathological, and biochemical findings.

There is no standard treatment for protothecosis. Many treatment strategies have been attempted, with variable clinical response [4]. A combination of medical and surgical approaches is most commonly used, and antifungal drugs, such as ketoconazole, itraconazole, fluconazole, conventional amphotericin B, and liposomal amphotericin B are the most commonly used antimicrobial agents [3]. Previous studies have shown that *Prototheca* species are normally susceptible to amphotericin B and variably susceptible to azoles [4, 18, 19]. The susceptibility to azoles could be explained by the presence of ergosterol in their cell membranes, and the absence of D-glucans in their cell walls could be the reason for resistance to echinocandins [19–21]. Our patient was successfully treated with excision biopsy and antifungal therapy, including fosfluconazole and topical amphotericin B, based on the MIC results. However, as there are no official guidelines and breakpoints for in vitro susceptibility testing of *Prototheca* species, it is difficult to interpret the results of disc diffusion zone diameters and MICs using automated systems or E-tests [22]. MIC testing is not always reproducible, and when it comes to treatment with azoles, in vitro susceptibility does not correlate with favorable clinical outcomes [4, 8]. It remains debatable how these results should be interpreted, but in vitro susceptibility testing could be helpful for choosing a better treatment regimen.

Here, we report a case of human protothecosis of the pharynx, and our results provide valuable information on the diagnosis and treatment of protothecosis in clinical practice.

## Data Availability

The data supporting the conclusions of this article are included within the article.

## Abbreviations

CT: Computed tomography
MALDI-TOF: Matrix-assisted laser desorption/ionization time-of-flight mass spectrometry
MIC: Minimum inhibitory concentration
PCR: Polymerase chain reaction
